# Clinicopathological features and prognosis of omental gastrointestinal stromal tumor: evaluation of a pooled case series

**DOI:** 10.1038/srep30748

**Published:** 2016-07-29

**Authors:** Fan Feng, Yangzi Tian, Zhen Liu, Shushang Liu, Guanghui Xu, Man Guo, Xiao Lian, Daiming Fan, Hongwei Zhang

**Affiliations:** 1 Department of Digestive Surgery, Xijing Hospital, Fourth Military Medical University, 127 West Changle Road, 710032, Xi’an, Shaanxi, China; 2 Department of Dermatology, Xijing Hospital, Fourth Military Medical University, 127 West Changle Road, 710032, Xi’an, Shaanxi, China

## Abstract

Clinicopathological features and prognosis of omental GISTs are limited due to the extremely rare incidence. Therefore, the aim of the present study was to investigate the clinicopathological features and prognosis of omental GISTs. Omental GISTs cases were obtained from our center and from case reports and clinical studies extracted from MEDLINE. Clinicopathological features and survivals were analyzed. A total of 99 cases of omental GISTs were enrolled in the present study. Omental GISTs occurred predominantly in greater omentum (78/99, 78.8%). The majority of tumors exceeded 10 cm in diameter (67/98, 68.3%) and were high risk (88/96, 91.7%). Histological type was correlated with tumor location and mutational status. The five year DFS and DSS was 86.3% and 80.6%, respectively. Mitotic index was risk factor for prognosis of omental GISTs. Prognosis of omental GISTs was worse than that of gastric GISTs by Kaplan-Meier analysis. However, multivariate analysis showed that the prognosis was comparable between the two groups. The majority of omental GISTs were large and high risk. Mitotic index was risk factor for prognosis of omental GISTs. The prognosis was comparable between omental and gastric GISTs.

Gastrointestinal stromal tumors (GISTs) are the most common mesenchymal tumor of the gastrointestinal tract^1^. GISTs are believed to originate from the interstitial cells of Cajal, the pacemaker cells of gastrointestinal tract^2^. GISTs can occur anywhere throughout the gastrointestinal tract. The most common locations are the stomach (40 to 70%), followed by small intestine (20 to 40%), and colorectum (5 to 15%)^3^. GISTs that arise outside the gastrointestinal tract as primary tumor are designated as extra-gastrointestinal stromal tumors (EGISTs). The EGISTs are located in the omentum, mesentery, liver, pancreas and retroperitoneum, etc^4^.

Studies involving large numbers of omental GISTs are extremely rare. To date, there was only one study contained a relatively large cases of omental GISTs^5^. Thus, various questions remain unanswered with respect to the clinicopathological features and prognosis. Therefore, the aim of the present study was to investigate the clinicopathological features and prognosis of omental GISTs.

## Results

The clinicopathological features were summarized in Table 1. There were 55 male (59.1%) and 38 female (40.9%). The patient age ranged from 22–99 years (median, 60 years; mean, 59.2 years). The most common symptom was abdominal pain (25/51, 49.0%), followed by abdominal mass (10/51, 19.6%) and abdominal distension (8/51, 15.7%). Twenty-one tumors located in the lesser omentum (21.2%), 78 tumors located in the greater omentum (78.8%). Eighty-six patients underwent complete surgical resection (86/95, 90.5%), 4 patients underwent palliative surgical resection (4/95, 4.2%), and 5 patients did not receive surgical resection (5/95, 5.3%).

The tumors ranged from 0.7 to 40 cm in maximum diameter (median, 13.0 cm; mean, 14.1 cm). Forty-two patients displayed spindle cell morphology (42/87, 48.3%), 29 patients displayed epithelioid morphology (29/87, 33.3%) and 16 patients displayed mixed morphology (16/87, 18.4%). The mitotic index of 32 patients exceeded 5/50 HPF (32/85, 37.6%). CD117 positivity was detected in 49 patients (49/58, 84.5%), CD34 positivity was detected in 36 patients (36/43, 83.7%) and DOG-1 positivity was detected in 8 patients (8/9, 88.9%). Twenty-seven patients were analyzed for gene mutation status. Nine patients carried KIT mutation (9/27, 33.3%), 14 patients carried PDGFRA mutation (14/27, 51.9%), the remaining 5 patients were wild type (5/27, 18.5%). According to NIH risk classification, 2 patients were very low risk (2/96, 2.1%), 5 patients were low risk (5/96, 5.2%), 1 patient was intermediate risk (1/96, 1.0%), and 88 patients were high risk (88/96, 91.7%). Information of adjuvant imatinib therapy were recorded in 58 patients, and 15 patients (25.9%) received imatinib therapy.

**Figure 1:**
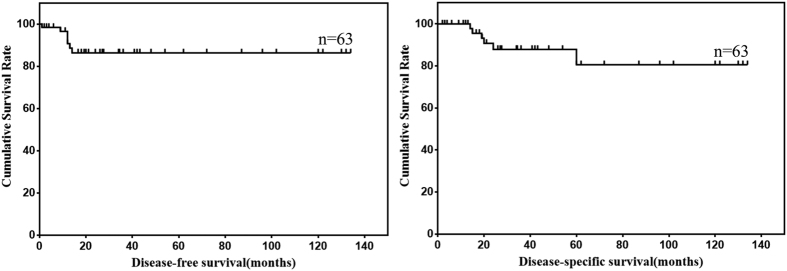
DFS and DSS of omental GISTs.

Survival data of omental GISTs were summarized in Table 1. Survival data of 63 patients were eventually selected for analysis using exclusion criteria described in the materials and methods. The follow up time ranged from 2 to 134 months (mean, 36.6 months; median, 21.1 months). Seven patients showed recurrence or metastasis, 6 patients suffered from GIST related deaths. The 1-, 3- and 5-year DFS was 90.8%, 86.3% and 86.3%, respectively. The 1-, 3- and 5-year DSS was 100.0%, 87.9% and 80.6%, respectively. The DFS and DSS of omental GISTs were analyzed using Kaplan-Meier survival analyses and shown in Fig. 1.

The relationship between clinicopathological features were analyzed (data not shown). The histological type was correlated with tumor location and mutational status. The ratio of epithelioid and mixed morphology of greater omental GISTs were significantly higher than that of lesser omental GISTs (P = 0.036). The epithelioid and mixed morphology were significantly correlated with PDGFRA mutation (P < 0.001).

**Figure 2:**
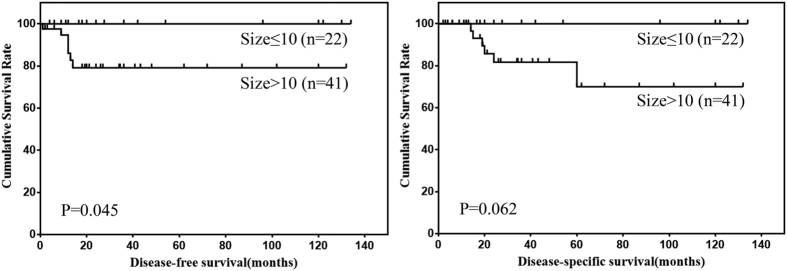
DFS and DSS of omental GISTs by tumor size.

**Figure 3:**
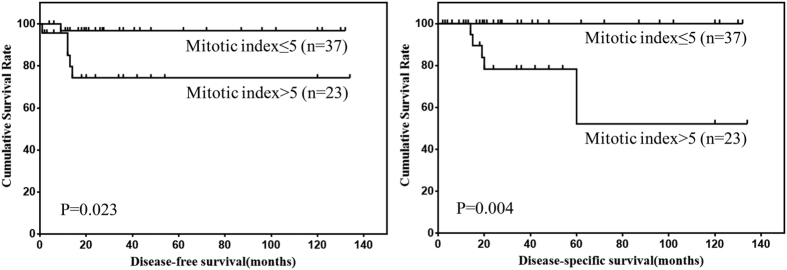
DFS and DSS of omental GISTs by mitotic index.

Prognostic factors for DFS and DSS of omental GISTs according to univariate analysis were shown in Table 2. The results showed that the tumor size and mitotic index were risk factors for DFS of omental GISTs, and mitotic index was the only risk factor for DSS of omental GISTs. The DFS and DSS of omental GISTs according to tumor size and mitotic index were shown in Figs 2 and 3.

The clinicopathological features of 99 omental GISTs including age, gender, tumor size, histological type, mitotic index and NIH risk category were compared with 297 gastric GISTs in our institution (Table 3). The results showed that the distribution of tumor size, histological type and NIH risk category were significantly different between omental and gastric GISTs (all with P < 0.001).

**Figure 4:**
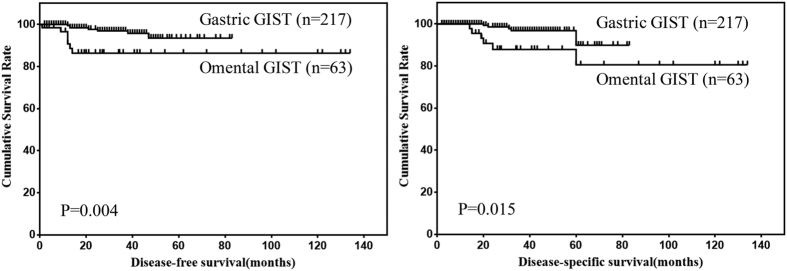
Comparison of DFS and DSS between omental and gastric GISTs.

In order to compare the prognosis of omental GISTs with gastric GISTs, survivals of 63 cases of omental GISTs and 217 cases of gastric GISTs with follow up data were analyzed. The results showed that the DFS and DSS of omental GISTs were significantly worse than that of gastric GISTs (Fig. 4). Further, multivariate analysis was performed to evaluate the prognostic value of locations (Table 4). The results showed that location was not an independent risk factor for prognosis of omental and gastric GISTs.

## Discussion

Clinicopathological features and prognosis of omental GISTs are limited due to the extremely rare incidence. Therefore, the aim of the present study was to investigate the clinicopathological features and prognosis of omental GISTs from our center and from literatures in MEDLINE. The present study represents the largest analysis of omental GISTs and indicates some features significantly associated with omental GISTs.

To date, the largest cases of omental GISTs was reported by Miettinen *et al.*^5^. The study contained 95 cases. However, it mainly focused on clinicopathological features of omental GISTs. The distribution of age, gender, tumor size, and mitotic index were similar to our present study. However, a few highlights with respect to clinicopathological features were revealed and the prognosis of omental GISTs were analyzed in depth in our present study.

The precise etiology of omental GISTs remains to be clarified. In the study reported by Miettinen *et al.*^5^, over half of the solitary omental GISTs were attached to or involved the gastrointestinal tract, and the histologic features were similar to gastric or small intestinal GISTs. Thus, they believed that solitary omental GISTs are actually externally extending gastric or small intestinal GISTs, and many others may have lost their original connection to the stomach or small intestine and become parasitically attached into the omentum. For multiple omental GISTs, they were believed to be metastatic tumors from an overlooked primary tumor.

However, it has been reported that GISTs in the omentum are derived from mesenchymal cells that are less differentiated than ICCs^6^. These may be ICC precursors straying into the abdominal cavity^7^. Moreover, Sakurai *et al.* found that KIT positive bipolar cells were present just beneath the mesothelial cells of the omentum. Thus, the identification of an ICC-counterpart in the omentum is the evidence that omental GISTs may also originate from ICC. They also demonstrated the existence of ICC-like cells focally in the omentum at 21 weeks of human gestation^8^. However, it is unknown whether they develop *in situ* or migrate from the ICC of the tubular GI tract at particular point in fetal development. Dedemadi *et al.* reported that there is no difference in the incidence between lesser and greater omentum^9^. However, the incidence of GISTs in the greater omentum was approximately four times as much as that of lesser omentum in our present study. The difference in the incidence between lesser and greater omentum needs further investigation.

In our present study, the majority of omental GISTs exceeded 10 cm in diameter and approximately ninety percent of the tumors were classified as high risk category. The spectrum of tumor size and NIH risk category of omental GISTs were significantly different from that of gastric GISTs in our institution. This may attribute to the absence of specific symptoms when the tumor was not large enough in the omentum or tumors did not invade adjacent gastrointestinal tract. Once the GISTs of the omentum reached a significant size, symptoms will appears including abdominal pain, mass, distension, fatigue and discomfort. Thus, early diagnosis of omental GISTs is very difficult.

Histologically, most GISTs display spindle cell morphology (70%), whereas a minority is of epithelioid (20%) or mixed phenotypes (10%)^10^. However, in the study reported by Miettinen *et al.*^5^, 53 out of 89 tumors showed spindle cell morphology (59.6%), 28 tumors were epithelioid (31.5%) and 8 tumors were mixed (8.9%). In our present study, 29 tumors displayed epithelioid morphology (33.3%) and 16 tumors displayed mixed morphology (18.4%). The proportion of epithelioid and mixed morphology of omental GISTs were significantly higher than that of gastric GISTs in our center and previous report. This indicated that the constituent ratio of epithelioid and mixed morphology could be various from each other depending on the location of GISTs. Further, we found that the incidence of epithelioid and mixed cell morphology was higher in greater omental GISTs than in lesser omental tumors. This may indicate that the origins of tumors in lesser omentum and greater omentum were different from each other, which needed further investigation.

In 1998, Hirota *et al.* reported their groundbreaking discovery of KIT mutations in GISTs. It is now established that 70% to 80% of GISTs harbor a KIT gene mutation^11^, and PDGFRA mutations occur in approximately 8% to 10% of gastric GISTs^12^. In the study reported by Miettinen *et al.*^5^, KIT mutation was detected in 15/36 tumors (41.7%), and PDGFRA mutation was detected in 11/36 tumors (30.6%). However, in our present study, only 9/27 tumors (33.3%) harbored KIT mutation but 14/27 tumors (51.9%) harbored PDGFRA mutation. Although the incidence of PDGFRA mutation in Miettinen’s and our report were higher than previous report, the results in our present study was even higher than that in Miettinen’s report. It was reported that spindle cell morphology correlates with KIT mutations^13^ and epithelioid and mixed cell morphology correlates with PDGFRA mutations^14^. This has also been demonstrated in our present study, the KIT mutations almost exclusively occurred in spindle cell morphology, and PDGFRA mutations almost exclusively occurred in epithelioid cell morphology. This indicated that KIT and PDGFRA mutant GISTs probably represent two distinct clinicopathological and molecular genetic disease entities. However, this needs further investigations in depth. It must be pointed out that the data of mutational analysis is only available in too few cases (27/99, 27.3%) in our present study, which are extremely too low to characterize the mutation spectrum of omental GISTs. The limited data could also result in bias during analyzing the association between cell morphology and mutational status. This was one limitation in our present study.

Besides tumor size and mitotic index, tumor location is also one important risk factor for the prognosis of GISTs^15^, and it was considered that extra-gastrointestinal stromal tumors were more aggressive than gastric GISTs in clinical course. However, the modified NIH risk classification system distinguishes only gastric from non-gastric GISTs, and the prognosis of omental GISTs are not discussed. Thus, we compared the prognosis of omental GISTs with gastric GISTs in our center. We found that the prognosis of omental GISTs was significantly worse than that of gastric GISTs. However, multivariate analysis showed that the prognosis was comparable between omental and gastric GISTs. This indicated that the prognosis of omental GISTs was as considerable as gastric GISTs. The significantly lower survival of omental GISTs than gastric GISTs in Kaplan-Meier survival analysis may attribute to the larger tumor size and higher NIH risk category of omental GISTs compared with gastric GISTs. However, it was inevitable that the extremely low incidence of imatinib therapy in our present study would result in bias during analysis of prognosis of omental GISTs. Thus, the actual disease free survival and disease specific survival of omental GISTs may be more favorable than that in our present study.

There are a few limitations in our present study. Firstly, the present study is a retrospective analysis and the completeness of data is limited. This will influence the analysis of clinicopathological features and prognosis. Secondly, the sample size of omental GISTs was not large enough, which will result in statistical bias. Thirdly, due to the limited sample size of duodenal and small intestinal GISTs in our center, the prognosis of omental GISTs were only compared to that of gastric GISTs.

## Conclusions

The majority of omental GISTs occurred in greater omentum, exceeded 10 cm in diameter and were high risk. The incidence of epithelioid cell morphology and PDGFRA mutation were relatively high in omental GISTs. The histological type was correlated with location and mutational status. Mitotic index was risk factor for prognosis of omental GISTs. Omental GISTs differ significantly from gastric GISTs in respect to clinicopathologic features. The prognosis was comparable between omental and gastric GISTs.

## Methods

GISTs cases of the omentum were from our institution and in addition from the literature. From May 2010 to March 2015, 2 cases of omental GISTs were diagnosed and treated in our institution. Literature search of MEDLINE was performed for all articles in English published from 1998 through 2015. MEDLINE search resulted in 47 case reports^8^,^9^,^16^,^17^,^18^,^19^,^20^,^21^,^22^,^23^,^24^,^25^,^26^,^27^,^28^,^29^,^30^,^31^,^32^,^33^,^34^,^35^,^36^,^37^,^38^,^39^,^40^,^41^,^42^,^43^,^44^,^45^,^46^,^47^,^48^,^49^,^50^,^51^,^52^,^53^,^54^,^55^,^56^,^57^,^58^,^59^,^60^ including 57 patients and 6 case series^61^,^62^,^63^,^64^,^65^,^66^ including 40 cases. As a result, a total of 99 omental GISTs patients were identified. In addition, the clinicopathological features and prognosis of 297 cases of gastric GISTs were compared with omental GISTs. This study was approved by the Ethics Committee of Xijing Hospital according to the provisions of the Declaration of Helsinki in 1995 (as revised in Edinburgh 2000)^67^, and written informed consent was obtained from the two patients in our center.

Data including age, gender, accompanied tumor, symptoms, location, tumor size, surgical intervention, histological type, immunohistochemical features, mutational status, mitotic index, NIH risk category, adjuvant therapy, tumor progression and survival data were recorded. The tumors were categorized into very low, low, intermediate and high risk groups according to the modified NIH risk classification criteria reported by Joensuu *et al.*^68^. For survival analysis, the inclusion criteria were listed as follows: 1. R0 resection, 2. without distant metastasis, 3. without GIST in other locations, 4. without other malignant tumors, 5. without neoadjuvant imatinib therapy, 6. with follow up data. Due to data acquisition, completeness of data is limited.

Data were processed using SPSS 22.0 for Windows (SPSS Inc., Chicago, IL, USA). Discrete variables were analyzed using the Chi-square test or Fisher’s exact test. Numerical variables were expressed as the mean ± SD unless. Significant predictors for survival identified by univariate analysis were further assessed by multivariate analysis. Evaluation of disease-free-survival (DFS) and disease-specific-survival (DSS) were obtained by the Kaplan-Meier method. The P values were considered to be statistically significant at the 5% level.

****Ethical approval and informed consent.**** This study was approved by the Ethics Committee of Xijing Hospital, and written informed consent was obtained from the two patients in our center.

## Additional Information

**How to cite this article**: Feng, F. *et al.* Clinicopathological features and prognosis of omental gastrointestinal stromal tumor: evaluation of a pooled case series. *Sci. Rep.* **6**, 30748; doi: 10.1038/srep30748 (2016).

## Figures and Tables

**Table 1:** Clinicopathological characteristics of 99 cases of omental GISTs.

Characteristics	Parameters
Age (∑ = 99)	
≤60	53 (53.5%)
>60	46 (46.5%)
Gender (∑ = 93)	
Male	55 (59.1%)
Female	38 (40.9%)
Accompanied tumor (∑ = 41)	
GISTs with other locations	7 (17.1%)
Other type of tumors	7 (17.1%)
Symptoms (∑ = 51)	
Abdominal pain	25 (49.0%)
Abdominal mass	10 (19.6%)
Abdominal distension	8 (15.7%)
Fatigue	5 (9.8%)
Abdominal discomfort	4 (7.8%)
Weight loss	4 (7.8%)
Location (∑ = 99)	
Lesser omentum	21 (21.2%)
Greater omentum	78 (78.8%)
Tumor size (∑ = 98)	
≤2 cm	3 (3.1%)
2.1–5 cm	9 (9.2%)
5.1–10 cm	19 (19.4%)
>10 cm	67 (68.3%)
Imaging features (∑ = 40)	
Solid	16 (40.0%)
Cystic	3 (7.5%)
Mixed	21 (52.5%)
Surgical resection (∑ = 95)	
Complete resection	86 (90.5%)
Incomplete resection	4 (4.2%)
No surgery	5 (5.3%)
Histological type (∑ = 87)	
Spindle	42 (48.3%)
Epithelioid	29 (33.3%)
Mixed	16 (18.4%)
Mitotic index (∑ = 85)	
≤5	53 (62.4%)
>5	32 (37.6%)
Immunohistochemisty	
CD117 (∑ = 58)	49 (84.5%)
CD34 (∑ = 43)	36 (83.7%)
DOG-1 (∑ = 9)	8 (88.9%)
Mutational status (∑ = 27)	
KIT	9 (33.3%)
PDGFRA	14 (51.9%)
Wild type	5 (18.5%)
NIH risk category (∑ = 96)	
Very low risk	2 (2.1%)
Low risk	5 (5.2%)
Intermediate risk	1 (1.0%)
High risk	88 (91.7%)
Adjuvant therapy (∑ = 58)	
Yes	15 (25.9%)
No	43 (74.1%)
Follow up time	
Mean (m, ±SD)	36.6 ± 36.4
Median (m, range)	21.1 (2, 134)
Survival data	
Recurrence or metastasis	7
GISTs related deaths	6
Survival rates (%)	
1-/3-/5-year DFS	90.8/86.3/86.3
1-/3-/5-year DSS	100.0/87.9/80.6

**Table 2:** Prognostic factors for DFS and DSS in patients with omental GISTs according to univariate analysis (n = 63).

Prognostic factors	β	Hazard ratio (95% CI)	P value
DFS			
Age (≤60/>60)	0.880	2.411(0.468–12.429)	0.293
Gender (male/female)	−0.190	0.827(0.160–4.269)	0.821
Location (lesser/greater omentum)	0.343	1.409(0.169–11.752)	0.751
Tumor size (≤10/>10)	3.724	41.440(0.067–25811.738)	0.045
Histological type(spindle/epithelioid/mixed)	0.393	1.482(0.569–3.863)	0.421
Mitotic index (≤5/>5)	2.082	8.021(0.937–68.653)	0.023
NIH risk category (1,2,3/4)	1.320	3.724(0.025–551.840)	0.605
DSS			
Age (≤60/>60)	0.688	1.989(0.364–10.868)	0.427
Gender (male/female)	0.168	1.183(0.217–6.462)	0.846
Location (lesser/greater omentum)	3.279	26.556(0.002–376917.747)	0.501
Tumor size (≤10/>10)	3.724	42.173(0.042–42476.817)	0.289
Histological type (spindle/epithelioid/mixed)	0.806	2.239(0.784–6.392)	0.132
Mitotic index (≤5/>5)	4.836	125.999(0.062–255451.941)	0.004
NIH risk category (1,2,3/4)	1.277	22.142(0.004–3225.535)	0.713

**Table 3:** Comparison of selected clinicopathological parameters between omental and gastric GISTs.

Characteristics	Omentum(n = 99)	Stomach(n = 297)	P value
Age			
≤60	53	168	0.599
>60	46	129	
Gender			
Male	55	155	0.241
Female	38	142	
Tumor size			
≤2 cm	3	96	<0.001
2.1–5 cm	9	107	
5.1–10 cm	19	72	
>10 cm	67	22	
Histological type			
Spindle	42	275	<0.001
Epithelioid	29	3	
Mixed	16	19	
Mitotic index			
≤5	53	163	0.221
>5	32	134	
NIH risk category			
Very low	2	83	<0.001
Low	5	58	
Intermediate	1	87	
High	88	69	

**Table 4:** Comparative survival analysis of omental and gastric GISTs using univariate and multivariate analysis.

Survival	Omentum	Stomach	Univariate analysis			Multivariate analysis		
	(n = 63)	(n = 217)	β	HR (95% CI)	P	β	HR (95% CI)	P
DFS			−1.407	0.245 (0.086–0.700)	0.004	0.033	1.033 (0.244–4.371)	0.965
1 year	90.8	99.5						
3 year	86.3	96.9						
5 year	86.3	93.5						
DSS			−1.399	0.247 (0.073–0.834)	0.015	0.013	1.013 (0.212–4.847)	0.987
1 year	100.0	100.0						
3 year	87.9	96.8						
5 year	80.6	89.9						
